# Chemical Diversity of Headspace and Volatile Oil Composition of Two Brown Algae (*Taonia atomaria* and *Padina pavonica*) from the Adriatic Sea

**DOI:** 10.3390/molecules24030495

**Published:** 2019-01-30

**Authors:** Igor Jerković, Marina Kranjac, Zvonimir Marijanović, Marin Roje, Stela Jokić

**Affiliations:** 1 Department of Organic Chemistry, Faculty of Chemistry and Technology, University of Split, Ruđera Boškovića 35, 21000 Split, Croatia; mkranjac@ktf-split.hr; 2 Department of Food Technology and Biotechnology, Faculty of Chemistry and Technology, University of Split, Ruđera Boškovića 35, 21000 Split, Croatia; zmarijanovic@ktf-split.hr; 3 Division of Organic Chemistry and Biochemistry, Ruđer Bosković Institute, Bijenička cesta 54, 10000 Zagreb, Croatia; mroje@irb.hr; 4 Department of Process Engineering, Faculty of Food Technology, Josip Juraj Strossmayer University of Osijek, Franje Kuhača 20, 31000 Osijek, Croatia; stela.jokic@ptfos.hr

**Keywords:** marine algae, headspace solid-phase microextraction (HS-SPME), hydrodistillation (HD), gas chromatography and mass spectrometry (GC-MS), sesquiterpenes, dimethyl sulfide, octan-1-ol

## Abstract

Two selected brown algae (*Taonia atomaria* and *Padina* *pavonica* from the family Dictyotaceae, order Dictyotales) growing in the same area (island Vis, central Adriatic Sea) were collected at the same time. Their phytochemical composition of the headspace volatile organic compounds (HS-VOCs; first time report) was determined by headspace solid-phase microextraction (HS-SPME). Hydrodistillation was applied for the isolation of their volatile oils (first report on *T. atomaria* volatile oil). The isolates were analyzed by gas chromatography (GC-FID) and mass spectrometry (GC-MS). The headspace and oil composition of *T. atomaria* were quite similar (containing germacrene D, epi-bicyclosesquiphellandrene, β-cubebene and gleenol as the major compounds). However, *P. pavonica* headspace and oil composition differed significantly (dimethyl sulfide, octan-1-ol and octanal dominated in the headspace, while the oil contained mainly higher aliphatic alcohols, *trans*-phytol and pachydictol A). Performed research contributes to the knowledge of the algae chemical biodiversity and reports an array of different compounds (mainly sesquiterpenes, diterpenes and aliphatic compounds); many of them were identified in both algae for the first time. Identified VOCs with distinctive chemical structures could be useful for taxonomic studies of related algae.

## 1. Introduction

Marine secondary metabolites possess outstanding structural and functional diversity related to their different metabolic pathways [1]. While the volatile organic compounds (VOCs) of terrestrial plants have attracted attention since antiquity, the VOCs of marine algae have been much less investigated. Therefore, the target of the present research are VOCs of two brown seaweeds from family Dictyotaceae, order Dictyotales: *Taonia atomaria* (Woodward) J. Agardh, 1848 and *Padina pavonica* (Linnaeus) Thivy, 1960.

*T. atomaria* (family Dictyotaceae, order Dictyotales, class Phaeophyceae) is a brown seaweed widespread in the Mediterranean Sea. Taondiol and atomaric acid, cyclised meroditerpenoids, were isolated from this alga collected in Canary Islands [2,3,4]. The chemical investigation of *T. atomaria* from the northern Adriatic Sea describes the identification of sesquiterpenes 4-cadinene, cadina-4(14),5-diene, (−)-germacrene D, axenol, (−)-cubebol and epi-cubebol from its petrol extract [5]. Two novel cyclised meroditerpenoids, atomarianones A and B, were isolated from MeOH/CH_2_Cl_2_ extract of *T. atomaria* collected at Serifos island in the Central Aegean Sea [6]. The freeze-dried alga *T. atomaria*, after exhaustive extraction with CH_2_Cl_2_/MeOH, followed by chromatographic separations, afforded the compounds of tetraprenyl toluoquinone class, e.g., (*Z*)- and (*E*)-sargaquinone and sargaol [7]. *T. atomaria* sulfolipids were characterised by high contents of palmitic acid and the main constituents were sulfoquinovosyl-di-acylglycerol and sulfoquinovosyl acylglycerol [8]. The extracts of *T. atomaria* exhibited: (a) anticancer activity due the presence of algal sulfolipids [8], the compounds stypodiol [9] and stypoldione were determined as in vitro inhibitors of microtubule polymerization [10]; (b) antifungal activity due to taondiol presence [11]; (c) antibacterial activity due to algal sulfolipids [8]; (d) insecticidal activity related to isoepitaondiol [12]; (e) antiviral activity related to the algal sulfolipids [8]. In addition, the extracts of *T. atomaria* exhibited the best radical-scavenging activity (RSA) using DPPH and chemiluminescence (CL) tests in comparison to the extracts of the other 12 algal species from the island Crete, Greece and approached the activity of powerful antioxidant standards [13]. The isolated metabolites taondiol, isoepitaondiol, stypodiol, stypoldione, sargaquinone and sargaol were found to possess marked RSA. Recently, it was found that the surface metabolites of *T. atomaria* exhibit the ability to regulate epibiosis [14].

*P. pavonica* belongs to the family Dictyotaceae, order Dictyotales, class Phaeophyceae and is widespread in the Adriatic Sea. The characteristic brown algal polysaccharides alginates and laminarans were found in *P. pavonica* [15]. Chlorophyll C_1_ and C_2_, fucoxanthin, fucoxanthol, flavoxanthin and diatoxanthin were the most typical pigments in *P. pavonica* [16]. The chemical composition of this seaweed from the southern Adriatic Sea was investigated [17] and 12 sterols were identified in CH_2_Cl_2_/MeOH extract, the main ones being cholesterol and fucosterol. The most abundant fatty acid was palmitic acid, followed by oleic acid and myristic acid. The toluene extract was subjected to 4-h simultaneous distillation-extraction (SDE; [17]); and in the volatile fraction, aromatic esters, benzyl alcohol, benzaldehyde, and free fatty acids dominated with low concentrations of terpenoids, phenols and sulfur containing compounds. *P. pavonica* collected from Red sea coast, Egypt was subjected to direct SDE and bis-2-ethylhexyl phthalate was the major compound. *P. pavonica* extracts exhibited: (a) antimicrobial activity [17,18]; (b) antifungal activity [19,20]; (c) antioxidant activity [20] that may be due to phenolic compounds; (d) cytotoxicity against KB cells [21]; oxysterol and hydroperoxy-24 vinyl-24 cholesterol were identified as being responsible for this activity; and (e) antitumor activity against lung (H460) and liver (HepG2) human carcinoma cell lines [22] due to xenicane diterpenes 18,19-epoxyxenic-19-methoxy-18-hydroxy-4-acetoxy-6,9,13-triene and 18,19-epoxyxenic-18,19-dimethoxy-4-hydroxy-6,9,13-triene.

Although there are few previous reports on the high-molecular VOCs from *T. atomaria* [5,23] and *P. pavonica* [17,22,24], there are no reports on the headspace composition of these algae. Headspace solid-phase microextraction (HS-SPME) is the appropriate method for the research of low-molecular and highly-volatile headspace compounds, while hydrodistillation (HD) is an adequate method for isolating volatile and less-volatile compounds. It is well known that the headspace composition is different form the hydrodistillate/extract composition, and certain lower molecular compounds can be found only in the headspace. Among them, chemotaxonomic markers, allelochemicals, defensive compounds, attractants, or alarming pheromones can be found that are participating in chemical communications in marine ecosystems or playing ecological roles. In addition, there is no data on *T. atomaria* direct hydrodistillate (HD) composition, there are only reports on EtOAc and petroleum extract composition containing several sesquiterpenes among other constituents. Therefore, the goals of the present study on two selected brown algae growing in the same area (island Vis, Adriatic Sea) and collected at the same time were to: (a) determine the phytochemical percentage (%) composition of the headspace VOCs (first time report) by HS-SPME followed by gas chromatography and mass spectrometry (GC-FID; GC-MS) analysis; (b) determine by GC-FID and GC-MS the chemical composition of their volatile oils isolated by hydrodistillation (first report on *T. atomaria* volatile oil); (c) compare the results of the corresponding headspace and volatile oil chemical composition; (d) discuss the obtained results in comparison to the literature data, particularly regarding the extracts composition or the algal volatile oils from different geographic areas; and (e) indicate possible biosynthetic formation pathways of the major identified compounds according to the literature data.

## 2. Results and Discussion

In order to investigate the chemical diversity of the headspace and volatile oil composition from two brown algae of the family Dictyotaceae, order Dictyotales collected from the Adriatic Sea from the same area (island Vis, Croatia), two complementary methods were used: headspace solid-phase microextraction (HS-SPME) and hydrodistillation (HD) followed by gas chromatography and mass spectrometry (GC-FID; GC-MS) analysis. In order to avoid the changes that could occur in VOCs from the native samples after a long time of collection or drying, both algae were investigated as fresh samples. Two fibres of different polarity were used for HS-SPME (Polydimethylsiloxane/Divinylbenzene (PDMS/DVB) and Divinylbenzene/Carboxen/Polydimethylsiloxane (DVB/CAR/PDMS)) to obtain more complete chemical profiles. The results pointed out striking differences between the chemical profiles for the headspace and volatile oil of the same algae (depending on the compounds molecular mass and volatility). It should be taken into consideration that the headspace composition does not reflect the composition percentage of the sample (only of the headspace) and hydrodistillation is not an adequate method for the isolation of water-soluble, high-molecular compounds and less volatile compounds such as diterpenes.

### 2.1. Headspace and Volatile Oil Composition of T. atomaria

The headspace of *T. atomaria* is dominated with germacrene D (32.06% (PDMS/DVB fibre); 27.89% (DVB/CAR/PDMS fibre)) and sesquiterpene bicyclic hydrocarbons of cadinane type skeleton epi-bicyclosesquiphellandrene (27.49%; 25.13%) followed by cadina-3,5-diene (2.45%; 3.60%), *trans*-cadina-1(6),4-diene (1.21%; 2.39%) and zonarene (2.25%; 2.53%). An array of other cadinane type structures with minor percentages were identified (Table 1). Another abundant compound with tricyclic sesquiterpene skeleton of cubebane type was β-cubebene (12.80%; 10.74%) with a minor percentage of its isomer α-cubebene (2.53%; 2.48%). In addition, axane type (bicyclic spiro[4,5]decane) sesquiterpene alcohol gleenol was found among major constituents (9.68%; 11.02%). Several compounds were found as minor constituents exclusively in the headspace (Table 1) such as: dimethyl sulfide, 2-ethylhex-1-yl acetate, bornyl acetate, γ-elemene, cyclosativene, α-ylangene, and β-patchoulene. The headspace composition of this algae is reported for the first time.

Germacrene D (22.24%), epi-bicyclosesquiphellandrene (20.83%) and gleenol (15.35%) were dominant compounds in the hydrodistillate as well. Smaller percentages of other cadinane types as well as selinane type sesquiterpenes were found (Table 1). However, in the distillate, several higher molecular compounds were found (that were not present in the headspace) such as diterpenes (pachydictyol A, cembra-4,7,11,15-tetraen-3-ol and isocembrol), higher aliphatic compounds (e.g., tridecanal or pentadecanal), sesquiterpenes (*trans*-α-bergamotene, α-amorphene, junenol, di-epi-1,10-cubenol, or δ-cadinol), trisnorsesquiterpene albene and *p*-xylene.

The major VOCs found in the headspace and oil are known to exhibit different biological activities. Germacrene D is involved in plant–insect interaction, acting as a pheromone on receptor neurones [25] and is also an important deterrent and insecticidal agent against different parasites such as mosquitos, aphids and ticks [26,27]. Germacrene D could be considered responsible for the cytotoxic activity of *Kundmannia sicula* (L.) D.C. essential oil being the main compound present in the oil (81.2%) [28]. This is in agreement with the results reported by Setzer et al. [29] where germacrene D resulted active against human breast adenocarcinoma (MDA-MB 231 and MCF-7), human ductal carcinoma (Hs 578T) and human hepatocellular carcinoma (Hep G2). Epi-bicyclosesquiphellandrene could be connected with antidermatophytic activity [30]. Gleenol exhibited the following biological activities: termiticidal, antihelminitic and growth regulation effects on plant seeds [31].

In comparison with previous research of *T. atomaria*, it is only possible to partially compare the results, since different extracts were investigated previously (not obtained by HS-SPME or HD, but with other methods targeting non-volatile compounds). The extracts mostly contained high-molecular compounds and researches were directed toward the isolation of individual components, not toward obtaining full chemical profiles. The compounds 4-cadinene, cadinane-4(14),5-diene, (−)-germacrene D, axenol, (−)-cubebol, and 4-epi-cubebol were previously identified in petroleum extract (after chromatographic purification) of dried *T. atomaria* collected in the North Adriatic sea [5]. Therefore, partial similarity with current research data is notable (the presence of cadinane type sesquiterpenes). Less similarity was noticed with respect to recent research on MeOH/CH_2_Cl_2_ extract of *T. atomaria* [14]. Germacra-4(15),5,10(14)-trien-9-ol was identified together with five other sesquiterpenes (−)-gleenol, α-cadinol methyl ether, (−)-*trans*-calamenene, (1*S*,5*E*,7*S*) 1-acetoxygermacra-4(15),5,10(14)-triene, and 4-peroxymuurol-5-ene. Moreover, two lipidic compounds, polyunsaturated fatty acid, (5*Z*,8*Z*,11*Z*,14*Z*,17*Z*)-eicosa-5,8,11,14,17-pentaenoic acid, and glycerol derivative, sn-3-*O*-(geranylgeranyl)glycerol were also isolated. However, in comparison with the mentioned results of other researchers, current research presents a complete chemical profile of the headspace and volatile oil composition, providing new data: epi-cyclosesquiphellandrene as a dominant compound was found for the first time in *T. atomaria* as well as numerous other sesquiterpenes with lower abundance (Table 1), not previously reported.

### 2.2. Headspace and Volatile Oil Composition of P. pavonica

A total of 17 compounds were found in the headspace of *P. pavonica*. Aliphatic fatty alcohol octan-1-ol was a dominant constituent (37.73%; 38.60%) along with its oxygenated derivative octanal (9.63%; 7.98%). Another major constituent was dimethyl sulfide (DMS; 18.27%; 26.37%). All three compounds were reported for the first time in this alga (only in the headspace). However, C_8_-hydrocarbons were previously found in various marine algae [32,33], usually (3*Z*,5*E*)-octa-1,3,5-triene (fucoserratene), but octan-1-ol was not found previously in such high headspace abundance. On the other hand, octan-1-ol was found in raw water taken during *Synura uvella* algal blooms after adsorption on Tenax and thermal desorption [34]. DMS, a highly-volatile organosulfur compound, serves as a chemoattractant for phytoplankton, bacteria, zooplankton, fish, and sea birds [35]. Three sulfur-containing compounds (methylethyl disulfide, diethyl disulfide and benzothiazole) were previously identified in toluene extract of *P. pavonica* that was subjected to 4-h simultaneous distillation-extraction (SDE) [17], but DMS was not present. Neither was it found after direct SDE of this algae [22]. Therefore, in accordance with our previous paper [32], we can use HS-SPME as the appropriate method for monitoring DMS presence. (*E*)-β-farnesene (7.92%; 6.28%) was a major sesquiterpene, followed by β-bisabolene, α-farnesene and *cis*- and *trans*-calamenene (Table 2). Smaller percentages of dictyopterene A and dictyopterene D′ were found. Benzaldehyde was found as a minor headspace constituent, and was also identified after SDE of *P. pavonica* toluene extract [17].

The chemical profiles of *P. pavonica* hydrodistillate and headspace are significantly different containing only three common compounds ((*E*)-β-farnesene, pentadecane and diisobutyl phthalate), but with different abundance (Table 2). The major oil compounds were higher aliphatic alcohols: (*Z*)-octadec-9-en-1-ol (25.68%), hexadecan-1-ol (17.29%), and (*Z*,*Z*)-octadeca-3,13-dien-1-ol (7.55%) along with minor percentages of tetradecan-1-ol, pentadecanal, hexadecan-2-one, pentadecan-1-ol, (*Z*)-hexadec-11-en-1-ol, (*E*)-hexadec-11-en-1-ol, octadecanal, and (*Z*,*Z*)-octadeca-9,12-dien-1-ol. Higher aliphatic esters were found as minor constituents such as: methyl 5,8,11,14-eicosatetraenoate, methyl 5,8,11,14,17-eicosapentaenoate and methyl 8,11,14,17-eicosatetraenoate. Diterpene alcohols *trans*-phytol (6.45%) and pachydictol A (6.03%) were among the relevant compounds in the oil with a minor abundance of isopachydictol A. Several sesquiterpenes were present: *trans*-α-bergamotene, epi-β-santalene, α-humulene, β-santalene, (*E*)-β-guaiene, β-bisabolene, and (*E*)-α-bisabolene. These sesquiterpenes as well as pachydictol A and isopachydictyol A were for the first time found in *P. pavonica*. In comparison with previous papers on VOCs, from this alga, only partial similarity is observed. Namely, the major compound of SDE isolate [22] was bis-2-ethylhexyl phtalate (40.22%), while in the current research, diisobutyl phthalate was found as a minor constituent. Fatty esters such as methyl eicosa-5,8,11,14-tetraenoate, methyl eicosa-5,8,11,14,17-tetraenoate and diterpene phytol were found previously in this alga [22] as well in the current research. The presence of free fatty acids was also reported [22]. Sesquiterpene compounds β-cubebene, germacrene D and santalol were previously detected in *P. pavonica* SDE isolate [22], but not in the present research where several other sesquiterpenes were found (Table 2). *P. pavonica* toluene extract subjected to 4-h SDE [17] provided esters benzyl acetate and benzyl formate as the major compounds. Besides the esters of benzoic acid, significant concentrations of their biogenetic precursors benzaldehyde and benzyl alcohol were found, but they were not identified in the isolated oil. Therefore, the assumption that these compounds can serve as biomarkers for these algae [17] is questionable. In addition, low concentrations of the common marine algal terpene dihydroactinidiolide and C_13_-norisoprenoid β-ionone were found previously, and in the obtained oil, diterpenes isopachydictyol A and pachydictol A as well as C_13_-norisoprenoid 3-oxo-α-ionol were present.

### 2.3. Possible Biosynthetic Origin of the Major Identified VOCs

**Figure 1:**
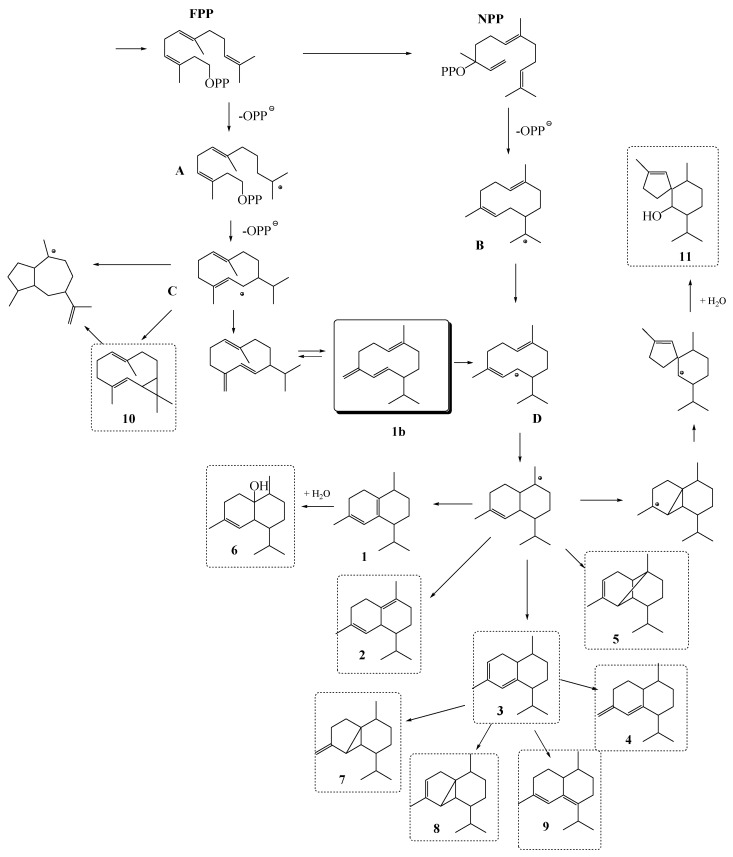
Possible biosynthetic correlations [37,38] among the main identified compounds in *T. atomaria*: **FPP**—farnesyl pyrophosphate; **NPP**—neryl pyrophosphate; germacreny cations (**A**, **B**, **C** and **D**); **1**: cadina-1(6),4-diene; **2**: δ-cadinene; **3**: cadina-3,5-diene; **4**: epi-bicyclosesquiphellandrene; **5**: ylangene; **6**: cubebol; **7**: β-cubebene; **8**: α-cubebene; **9**: zonarene; **10**: bicyclogermacrene; **11**: gleenol; **1b**: germacrene D.

Sesquiterpenes, dominant in the *T. atomaria* headspace and volatile oil, are C_15_-compounds containing the assembly of three isoprenoid units. The large number of sesquiterpenoid carbon skeletons arises from the common precursor, farnesyl pyrophosphate (FPP), by various modes of cyclizations usually followed by skeletal rearrangement (Figure 1). 

Selinane and cadinane are two main types of sesquiterpenes reported in brown algae [36]. According to the currently accepted hypotheses, in addition to farnesyl pyrophosphate (FPP), neryl pyrophosphate (NPP) may be a precursor in sesquiterpenes formation (Figure 1). It can be assumed [37] that in the cyclisation process (after pyrophosphate (PP) loss) germacrenyl cations A and B are formed, and after hydride migration, cations C and D are formed (Figure 1). For the biosynthesis of cadinene-type sesquiterpenes, two alternative pathways were suggested [38]. The primarily formed cation A may be transformed to cation C and after deprotonated to germacrene D (1b). The subsequent change from *cis*- structure to *trans*- 1b is crucial for the formation of cadinane type sesquiterpenes (with *Z* double bond in the ring) followed by protonation and rearrangement to cation D. As an alternative, NPP may serve as a substrate for cadinene-type sesquiterpene biosynthesis. In this case, the cyclization to the cadinanes would proceed via cations B and D. Moreover, it can be assumed that germacrene D and germacrenyl cation D are the biosynthetic precursors of the major isolated sesquiterpenes from *T. atomaria*. Major sesquiterpenes of this alga with cadinane skeleton that could be derived from germacrene D were (Figure 1): cadina-1(6),4-diene, δ-cadinene, cadina-3,5-diene, 4-epi-bicyclosesquiphellandrene; ylangene; cubebol, β-cubebene, α-cubebene, zonarene, and gleenol. Bicyclogermacrene is probably derived from germacrenyl cation C.

Dimethyl sulfide (DMS), a major compound of the *P. padina* headspace, results from an enzymatic decomposition of dimethyl-β-propiothetin [33], a metabolite of methionine that is fairly widespread in marine plants. Formed dimethylsulfoniopropionate (DMSP), a tertiary sulfonium compound, is the precursor of DMS. Recently, the algal enzyme responsible for the formation of DMS from DMSP was found and characterised in alga *Emiliania huxleyi* [39].

In general, the overall mechanism of enzymatic lipid degradation is identical in terrestrial plants [40] and algae [41]. The enzyme cascade is initiated by activated phospholipase, followed by lipoxygenase and hydroperoxide lyase. However, the particular enzymes are highly species- and sometimes even strain-specific [41], and this can explain the large biodiversity of lipid degradation VOCs (e.g., carbonyl compounds, alcohols, and hydrocarbons). Marine algae contain unsaturated fatty acids, and they can produce C_18_, C_20_ and C_22_ fatty acid hydroperoxides. Following the general concept of lipid peroxidation, and subsequent oxidative cleavage of the carbon skeleton, the biosynthesis of C_8_-hydrocarbons from *P. pavonica* could start from the polyunsaturated fatty acid substrate that could be activated [42] either by 9-lipoxygenase or by 12-lipoxygenase producing 9- or 12-hydroperoxides that further cleave oxidatively to produce C_8_-compounds. Octanal could originate from ω9 mono-unsaturated fatty acids (MUFAs) and also from ω6 poly-unsaturated fatty acids (PUFAs) such as linoleic acid [43].

## 3. Materials and Methods 

The samples of two brown algae *Taonia atomaria* (Woodward) J. Agardh, 1848 and *Padina pavonica* (Linnaeus) Thivy, 1960) were collected in the middle of the Adriatic Sea, in island Vis bay near Prirovo peninsula in May 2018. The geographical coordinates of the sampling were: 43°3′47″ N, 16°11′15″ E (*P. pavonica*) and 43°3′44″ N, 16°11′16″ E (*T. atomaria*). Single point sample collection provided representative samples. The algae were collected from depths of 1 m with the sea temperature at 17 °C. The samples were separately collected and placed in air-tight plastic bags containing surrounding seawater and were immediately transported to the laboratory. The samples were kept in the dark at 4 °C, and the extractions were performed within 48 h of the collection. Before headspace solid-phase microextraction (HS-SPME), each sample was separately cut into small pieces and the excess seawater was removed by placing it between the filter paper layers for 2 min (the seawater was not removed completely) as was done in previous research [32].

### 3.1. Headspace Solid-Phase Microextraction (HS-SPME)

Headspace solid-phase microextraction (HS-SPME) was performed with a manual SPME holder using two fibres covered with PDMS/DVB (Polydimethylsiloxane/Divinylbenzene) or DVB/CAR/PDMS (Divinylbenzene/Carboxen/Polydimethylsiloxane) obtained from Supelco Co. (Bellefonte, PA, USA). The fibres were conditioned prior to the extraction according to the instructions by Supelco Co. For HS-SPME, prepared samples (1 g) were placed separately in 5 mL glass vials and hermetically sealed with PTFE/silicone septa. The vials were maintained in a water bath at 60 °C during equilibration (15 min) and HS-SPME (45 min). After the sampling, the SPME fibre was withdrawn into the needle, removed from the vial, and inserted into the injector (250 °C) of GC-FID and GC-MS for 6 min where the extracted volatiles were thermally desorbed directly to the GC column. The procedure was similar as in previous paper [32]. HS-SPME was done in triplicate for each alga.

### 3.2. Hydrodistillation (HD)

Hydrodistillation was performed in a modified Clevenger apparatus for 2 h with the use of 1 mL of solvent trap (pentane:diethyl ether 1:2 *v*/*v*). The prepared samples (10 g; cut into small pieces) were used separately for the hydrodistillation. The volatile oil dissolved in the solvent trap was removed with a pipette, passed through the layer of MgSO_4_ in a small glass funnel and carefully concentrated by the slow flow of nitrogen until the volume of 0.2 mL. The hydrodistillation for each sample was performed in triplicate. One microlitre was used for GC-FID and GC-MS analyses.

### 3.3. Gas Chromatography and Mass Spectrometry (GC-MS) Analyses

Gas chromatography and mass spectrometry (GC-MS) analyses were done on an Agilent Technologies (Palo Alto, CA, USA) gas chromatograph model 7890A equipped with a flame ionization detector (FID) and a HP-5MS capillary column (5% phenyl-methylpolysiloxane, Agilent J and W). The GC conditions were the same as described previously [32]. In brief, the oven temperature was set up isothermally at 70 °C for 2 min, then increased from 70–200 °C at 3 °C·min^−1^, and held isothermally at 200 °C for 15 min; the carrier gas was helium (He 1.0 mL·min^−1^). The GC-MS analyses were done on an Agilent Technologies (Palo Alto, CA, USA) gas chromatograph model 7820A equipped with a mass selective detector (MSD) model 5977E (Agilent Technologies) and HP-5MS capillary column, under the same conditions as for the GC-FID analysis. The MSD (EI mode) was operated at 70 eV, and the mass range was 30–300 amu.

The identification of VOCs was based on the comparison of their retention indices (RI), determined relative to the retention times of *n*-alkanes (C_9_–C_25_), with those reported in the literature (National Institute of Standards and Technology [44]) and their mass spectra with the spectra from Wiley 9 (Wiley, New York, NY, USA) and NIST 14 (d-Gaithersburg) mass spectral libraries. The percentage composition of the samples was computed from the GC peak areas using the normalization method (without correction factors). The average component percentages in Table 1 and Table 2 were calculated from GC-FID and GC-MS analyses.

## 4. Conclusions

HS-SPME and hydrodistillation were adequate and complementary methods for the research of headspace and volatile oil composition of *T. atomaria* and *P. pavonica*. Although these two seaweed species belong to the same botanical family and order, and were collected from the same area at the same time, significant diversity in their VOCs composition was found. The headspace and oil composition of *T. atomaria* were quite similar (containing germacrene D, epi-bicyclosesquiphellandrene, β-cubebene and gleenol as the major compounds). However, the headspace and oil composition of *P. pavonica* differed significantly (dimethyl sulfide, octan-1-ol and octanal dominated in the headspace, while the oil contained mainly higher aliphatic alcohols, *trans*-phytol and pachdityol A). The current research contributed to the knowledge of algae chemical biodiversity since the obtained chemical profiles reveal an array of different compounds (mainly sesquiterpenes, diterpenes and aliphatic compounds); many of them were identified in both algae for the first time. Identified VOCs with distinctive chemical structures (among them biologically active compounds can be found) could be useful for algae taxonomic studies.

## Figures and Tables

**Table 1:** Headspace and volatile oil composition of *T. atomaria* (RI from the literature used for identifying the compounds).

No.	Compound	RI	Area Percentages (%)		
			HS-SPME (PDMS/DVB) ± SD	HS-SPME (DVB/CAR/PDMS) ± SD	HD ± SD
1	Dimethyl sulfide ^S^	523	0.06 ± 0.01	0.02 ± 0.01	-
2	*p*-Xylene ^S^	870	-	-	0.01 ± 0.01
3	2-Ethyl-1-hexyl acetate	1151	0.03 ± 0.01	0.03 ± 0.00	-
4	Albene	1160	-	-	0.01 ± 0.00
5	Bornyl acetate ^S^	1286	-	0.01 ± 0.00	-
6	γ-Elemene	1341	0.06 ± 0.01	0.09 ± 0.01	-
7	α-Cubebene ^S^	1353	2.53 ± 0.06	2.48 ± 0.05	0.16 ± 0.01
8	Cyclosativene ^S^	1368	0.01 ± 0.00	0.01 ± 0.00	-
9	α-Ylangene	1374	0.02 ± 0.00	0.06 ± 0.01	-
10	α-Copaene ^S^	1379	0.04 ± 0.01	0.12 ± 0.02	0.02 ± 0.00
11	β-Patchoulene	1383	0.02 ± 0.00	0.02 ± 0.01	-
12	β-Bourbonene	1387	0.71 ± 0.04	0.63 ± 0.02	0.49 ± 0.01
13	β-Cubebene	1393	12.80 ± 0.53	10.74 ± 0.01	6.10 ± 0.25
14	β-Ylangene	1421	0.50 ± 0.03	0.61 ± 0.04	0.17 ± 0.01
15	β-Copaene	1432	0.31 ± 0.02	0.45 ± 0.03	0.12 ± 0.01
16	*trans*-α-Bergamotene	1437	-	-	0.01 ± 0.00
17	Aromandendrene ^S^	1442	0.01 ± 0.00	0.02 ± 0.01	-
18	Isogermacrene D	1447	0.10 ± 0.02	0.13 ± 0.02	0.03 ± 0.01
19	Cadina-3,5-diene	1454	2.45 ± 0.07	3.60 ± 0.02	0.39 ± 0.02
20	(*E*)-β-Farnesene ^S^	1461	0.04 ± 0.00	-	0.02 ± 0.00
21	*cis*-Muurola-4(15),5-diene	1467	-	-	0.07 ± 0.01
22	α-Muurolene	1470	-	-	0.01 ± 0.00
23	Alloaromadendrene ^S^	1464	0.13 ± 0.02	0.26 ± 0.03	0.19 ± 0.02
24	γ-Gurjunene ^S^	1473	-	-	0.11 ± 0.01
25	*trans*-Cadina-1(6),4-diene	1477	1.21 ± 0.02	2.39 ± 0.04	-
26	α-Amorphene	1479	-	-	0.17 ± 0.02
27	Germacrene D ^S^	1486	32.06 ± 2.16	27.89 ± 2.00	22.24 ± 1.92
28	γ-Muurolene	1487	-	-	0.11 ± 0.02
29	epi-Bicyclosesquiphellandrene	1496	27.49 ± 2.03	25.13 ± 2.20	20.83 ± 1.90
30	γ-Amorphene	1495	-	-	2.10 ± 0.05
31	Bicyclogermacrene	1498	0.87 ± 0.05	0.63 ± 0.03	-
32	Epizonarene	1500	-	0.66 ± 0.08	-
33	α-Muurolene	1502	0.27 ± 0.02	0.57 ± 0.06	0.33 ± 0.04
34	β-Cadinene	1509	0.14 ± 0.01	0.22 ± 0.03	-
35	Tridecanal ^S^	1511	0.05 ± 0.01	0.01 ± 0.00	0.08 ± 0.01
36	γ-Cadinene	1517	0.40 ± 0.02	0.97 ± 0.05	2.14 ± 0.09
37	Cubebol	1519	1.08 ± 0.02	1.10 ± 0.01	-
38	δ-Cadinene ^S^	1527	1.13 ± 0.03	2.14 ± 0.05	1.09 ± 0.02
39	Zonarene	1529	2.25 ± 0.05	2.53 ± 0.08	1.45 ± 0.05
40	*trans*-Cadina-1,4-diene	1536	0.46 ± 0.02	0.83 ± 0.04	0.24 ± 0.01
41	α-Cadinene	1541	0.18 ± 0.01	0.49 ± 0.03	0.03 ± 0.01
42	α-Calacorene	1546	0.01 ± 0.00	0.08 ± 0.01	-
43	Gleenol	1590	9.68 ± 0.80	11.02 ± 0.70	15.35 ± 0.95
44	Junenol	1518	-	-	0.17 ± 0.02
45	Di-epi-1,10-cubenol	1629	-	-	0.54 ± 0.01
46	T-Cadinol	1646	0.31 ± 0.05	0.39 ± 0.04	1.59 ± 0.05
47	α-Muurolol (Torreyol)	1648	-	-	0.49 ± 0.05
48	δ-Cadinol	1654	-	-	0.25 ± 0.02
49	α-Cadinol	1659	0.03 ± 0.00	0.01 ± 0.00	1.45 ± 0.09
50	Pentadecanal	1714	0.03 ± 0.00	0.01 ± 0.00	0.16 ± 0.01
51	4,10(14)-Cadinadien-8-β-ol *	1784	1.03 ± 0.05	0.84 ± 0.08	1.94 ± 0.09
52	Diisobutyl phthalate ^S^	1870	0.01 ± 0.00	0.09 ± 0.01	4.23 ± 0.09
53	Isocembrol (Thunbergol)	2040	-	-	0.73 ± 0.02
54	Pachydictyol A	2123	-	-	3.26 ± 0.06
55	Cembra-4,7,11,15-tetraen-3-ol *	2226	-	-	2.46 ± 0.05

RI retention indices relative to C_9_–C_25_ alkanes, * tentatively identified, ^S^ identification confirmed with standard compound, SD standard deviation.

**Table 2:** Headspace and volatile oil composition of *P. pavonica*.

No.	Compound	RI	Area Percentages (%)		
			HS-SPME (PDMS/DVB) ± SD	HS-SPME (DVB/CAR/PDMS) ± SD	HD ± SD
1	Dimethyl sulfide ^S^	523	18.27 ± 1.46	26.37 ± 2.10	-
2	*p*-Xylene ^S^	869	-	-	0.06 ± 0.01
3	Benzaldehyde ^S^	970	2.48 ± 0.07	0.33 ± 0.02	-
4	6-Methylhept-5-en-2-one	990	0.87 ± 0.01	-	-
5	Octanal ^S^	1005	9.63 ± 0.91	7.98 ± 0.20	-
6	1,8-Cineole ^S^	1038	2.43 ± 0.05	0.87 ± 0.04	-
7	2-Ethylhexan-1-ol	1039	-	0.87 ± 0.03	-
8	2,2,6-Trimethylcyclohexanone ^S^	1041	0.68 ± 0.02	0.55 ± 0.01	-
9	Octan-1-ol ^S^	1079	37.73 ± 2.80	38.60 ± 2.71	-
10	α-Cumyl alcohol	1092	-	1.71 ± 0.05	-
11	Dictyopterene A	1119	1.27 ± 0.02	0.87 ± 0.03	-
12	Dictyopterene D′	1157	1.15 ± 0.05	-	-
13	Berkheyaradulene	1387	-	-	0.18 ± 0.01
14	β-Ylangene	1421	-	-	0.10 ± 0.00
15	*trans*-α-Bergamotene	1439	-	-	0.05 ± 0.01
16	epi-β-Santalene	1449	-	-	0.16 ± 0.03
17	α-Humulene	1455	-	-	0.05 ± 0.01
18	(*E*)-β-Farnesene ^S^	1460	7.92 ± 0.85	6.28 ± 0.99	0.03 ± 0.01
19	β-Santalene	1462	-	-	0.04 ± 0.01
20	(*E*)-β-Guaiene	1487	-	-	0.24 ± 0.02
21	epi-Bicyclosesquiphellandrene	1496	-	-	0.04 ± 0.00
22	Pentadecane ^S^	1500	4.30 ± 0.10	3.56 ± 0.09	1.11 ± 0.20
23	β-Bisabolene	1509	-	-	0.08 ± 0.00
24	α-Farnesene	1510	4.43 ± 0.11	-	-
25	*cis*-Calamenene	1526	-	0.62 ± 0.09	-
26	*trans*-Calamenene	1528	-	0.19 ± 0.02	-
27	(*E*)-α-Bisabolene	1544	-	-	0.01 ± 0.00
28	Tetradecanal ^S^	1613	-	-	0.10 ± 0.02
29	3-Oxo-α-ionol	1655	-	-	0.76 ± 0.09
30	Tetradecan-1-ol ^S^	1680	-	-	0.26 ± 0.03
31	Pentadecanal	1714	-	-	0.13 ± 0.01
32	Hexadecan-2-one ^S^	1772	-	-	0.23 ± 0.03
33	Pentadecan-1-ol ^S^	1782	-	-	0.10 ± 0.01
34	6,10,14-Trimethylpentadecan-2-one ^S^	1846	-	-	0.10 ± 0.00
35	(*Z*)-Hexadec-11-en-1-ol	1862	-	-	1.15 ± 0.08
36	Diisobutyl phthalate ^S^	1870	-	0.24 ± 0.02	2.15 ± 0.03
37	(*E*)-Hexadec-11-en-1-ol	1870	-	-	2.58 ± 0.05
38	Hexadecan-1-ol ^S^	1882	-	-	17.29 ± 2.50
39	Isopachydictol A	1889	-	-	0.15 ± 0.01
40	Octadecanal ^S^	2019	-	-	0.09 ± 0.00
41	Methyl 5,8,11,14-eicosatetraenoate (Methyl arachidonate) *	2035	-	-	0.66 ± 0.02
42	Methyl 5,8,11,14,17-eicosapentaenoate *	2039	-	-	3.98 ± 0.09
43	Methyl 8,11,14,17-eicosatetraenoate *	2045	-	-	1.84 ± 0.08
44	(*Z*,*Z*)-Octadeca-9,12-dien-1-ol	2051	-	-	3.60 ± 0.03
45	(*Z*)-Octadec-9-en-1-ol ^S^	2058	-	-	25.68 ± 2.26
46.	(Z,Z)-Octadeca-3,13-dien-1-ol *	2070	-	-	7.55 ± 0.09
47	*trans*-Phytol ^S^	2112	-	-	6.45 ± 0.08
48	Pachydictol A	2123	-	-	6.03± 0.09

RI retention indices relative to C_9_–C_25_ alkanes, * tentatively identified, ^S^ identification confirmed with standard compound, SD standard deviation.
